# Climate change and Indigenous mental health in the Circumpolar North: A systematic review to inform clinical practice

**DOI:** 10.1177/13634615211066698

**Published:** 2022-01-06

**Authors:** Laurence Lebel, Vincent Paquin, Tiff-Annie Kenny, Christopher Fletcher, Lucie Nadeau, Eduardo Chachamovich, Mélanie Lemire

**Affiliations:** 1 Population Health and Optimal Health Practices Axis, 36896 CHU de Québec-Université Laval Research Centre, Québec, QC, Canada; 2 Department of Social and Preventive Medicine, 4440 Université Laval, Québec, QC, Canada; 3 Douglas Mental Health Institute, Montréal, QC, Canada; 4 Department of Psychiatry, McGill University, Montréal, QC, Canada; 5 Montreal Children's Hospital, Montréal, QC, Canada

**Keywords:** Arctic regions, climate change, indigenous peoples, mental health, resilience

## Abstract

Climate change is disproportionally impacting the Circumpolar North, with particular impacts among Indigenous populations. Environmental changes are felt in many aspects of daily life of Northern communities, including both physical and mental health. Thus, health institutions from around the Arctic must meet emerging needs, while the phenomenon remains marginal to their southern counterparts. In this systematic review, we aimed to review current scientific knowledge on the mental health impacts of climate change in Indigenous Peoples across the Circumpolar North. Seven databases were searched. Original peer-reviewed research articles were included if they addressed links between climate change and mental health in Arctic or Subarctic Indigenous Populations. After extraction, data were synthesized using thematic analysis. Of the 26 articles that met inclusion criteria, 16 focused on Canadian Inuit communities and 21 were exclusively qualitative. Being on the land was identified as a central determinant of wellbeing. Immediate impacts of climate change on mental health were felt through restricted mobility and disrupted livelihoods. Effects on mental health were further felt through changes in culture and identity, food insecurity, interpersonal stress and conflicts, and housing problems. Various ways in how communities and individuals are coping with these effects were reported. Understanding climate-related pathways of mental health risks in the Arctic is crucial to better identify vulnerable groups and to foster resilience. Clinicians can play a role in recognizing and providing support for patients affected by these disruptions. Policies sensitive to the climate–mental health relationship must be advocated for.

## Introduction

Around the globe, the Circumpolar North is among the regions most affected by climate change (AMAP, 2017; Bell & Brown, 2018; IPCC, 2018, 2019). This area, which is the traditional and contemporary homeland of several Indigenous Peoples (the Circumpolar Peoples), comprises: Artic and Subarctic regions in Canada, inhabited by Inuit, Athabaskan, and Algonquian peoples; Alaska in the United States, home to multiple peoples including the Yupik, Inupiat, Aleut, and Athabaskan; Greenland, inhabited by the Inuit; the Sápmi region in Northern Europe (including parts of Sweden, Finland, and Norway), homeland to the Saami; and Russia, inhabited by many peoples including the Yakut and Yupik (Fardon et al., 2012). In Canada, Inuit communities are concentrated in the Inuvialuit Settlement Region (Northwest Territory and Yukon), Nunavut, Nunavik (Quebec), Nunatsiavut (Labrador), and NunatuKavut (Labrador). The presence of Indigenous peoples ranges between minority in Nordic countries, to up to 85% of the population in Nunavut and Greenland (Young et al., 2012).

The Circumpolar North has undergone rapid transformations since modern times: colonization, assimilation attempts, resource exploitation, environmental contamination, and immigration from southern states have impacted the social structures, livelihoods, and wellbeing of Northern communities (Csonka & Schweitzer, 2004; Kelman & Næss, 2019; Lehti et al., 2009; Young et al., 2012). Some of the early historical events include: the immigration of southern farmers into Saami territory in the 14th and 15th centuries; the arrival of European missionaries in Greenland (1721) and Arctic Canada (1860s); and the assignation of reindeer herds to collective farms in Siberia (1920–1940s) (Young et al., 2012). Assimilation efforts intensified in the 1950s and onwards, for example in Canada, where the government enforced the sedentarization of Northern Indigenous peoples. Several Inuit communities were displaced to more northernly areas, and dogs for transportation were slaughtered (Young et al., 2012). Indigenous children were put into residential schools where European customs, diet, and beliefs were imposed, and where they were subjected to physical, emotional, and sexual abuse (Csonka & Schweitzer, 2004; Kirmayer, 1994; Young et al., 2012).

Nowadays, there are socioeconomic and health disparities between Indigenous and non-Indigenous peoples across the Circumpolar North, with regional variations. Compared to settler populations, these inequalities generally include lower educational attainment (particularly in Northern Canada, and less so in Scandinavian countries), lower life expectancy, and lower income (Schmidt et al., 2015; Young et al., 2012), and higher rates of substance use, alcohol use, and suicide (Lehti et al., 2009; Young et al., 2012). The economy of the Circumpolar North is mainly resource-based, with domination by oil/gas in Alaska and Arctic Russia, important contributions of mining in Northern Canada, and contributions of hydroelectric power, manufacturing, and other technologies in Northern Scandinavian regions (Kelman & Næss, 2019; Schmidt et al., 2015; Young et al., 2012). Gains in political independence and land settlements have been made by Indigenous peoples, notably in Alaska, Northern Canada, Greenland, and Sàpmi in the 1970s and after (Csonka & Schweitzer, 2004; Young et al., 2012). In many communities, despite these socioeconomic transformations, the importance of subsistence activities for culture, identity, diet, and community wellbeing has been maintained, but often with concerns for their perennity and intergenerational transmission (Condon et al., 1995; Kelman & Næss, 2019; Kral et al., 2011).

The various land- and sea-based activities practiced by Circumpolar Peoples hold an important role in their spiritual, physical, and mental health (Boulanger-Lapointe et al., 2019; Cunsolo et al., 2020; Cunsolo Willox et al., 2012, 2015; Kirmayer et al., 2009; Sakakibara, 2009). Because of their intricate relationships with local geographies, flora, and fauna, and because of the rapidity of the climate change in the North, these diverse peoples are likely to be among those most affected by climate change (IPCC, 2014). Different patterns of subsistence activities based on local animal resources are practiced by Circumpolar Peoples, including maritime hunting (e.g., Inuit, coastal Chukchi), Taiga hunting and fishing (e.g., Dene), and reindeer herding (e.g., Sami, Chukchi) (Kelman & Næss, 2019; Young et al., 2012). They are all witnessing important disruptions of their environment including warmer temperatures, changes in animal behaviors and distribution, loss of land and sea ice, thawing permafrost, vegetation shifts, coastal erosion, and changes in the water cycle (AMAP, 2017; Bell & Brown, 2018; IPCC, 2014, 2019; Overland et al., 2019; Vincent, 2020).

Emerging research testifies to the direct and indirect impacts of these rapid transformations on the health of Circumpolar Peoples, encompassing weather-related accidents, vector-borne and other infectious diseases, food insecurity, and psychosocial disruptions (Cunsolo et al., 2020; Cunsolo Willox et al., 2012, 2015; Dudley et al., 2015; Furgal & Seguin, 2006; Waits et al., 2018). Recent evidence has brought to the fore several possible pathways linking climate change to mental health in the Circumpolar North (Cunsolo Willox et al., 2015; Middleton et al., 2020b). The environmental upheavals related to climate change are compounded by colonization and intergenerational trauma, with Indigenous Peoples being again dispossessed from their environment due to external, human-driven changes (Cunsolo Willox et al., 2013a; Furberg et al., 2011; Harper et al., 2015; Middleton et al., 2020b).

Communities, policymakers, and health institutions of Northern Canada and from around the Arctic have had limited resources to address the mental health care needs emerging from environmental changes. The vast majority of Northern communities are remote and supported by mental health professionals (psychologists and psychiatrists) who fly in for a limited period or use telehealth. Mental health care is also delivered in person by community workers, general practice nurses, and social workers. Most health care professionals are from settler cities, often outside the Circumpolar North, and receive little to no training to provide culturally relevant care in the North (Paquin et al., 2019). Thus, mental health in the North is shaped by unique cultural, ecological, socio-political, and historical factors, including the ongoing impacts of colonization. In this context, knowledge-sharing between communities, researchers, and health care providers is essential to mobilize all sectors around problem-solving approaches that acknowledge community-specific preoccupations and values (Paquin et al., 2020; Webb et al., 2010). This knowledge-sharing process can be facilitated through literature reviews that are usable by health practitioners and that synthesize research on the perspectives, needs, and experiences of Northern communities (Paquin et al., 2019).

The current study thus aimed: (1) to systematically review scientific literature on the effects of climate change on the mental health of Circumpolar Indigenous Peoples, (2) to explore how these populations are reacting to and coping with these effects, and (3) to comment on their implications for clinical practice.

## Methods

### Study design

We conducted a systematic review of the scientific literature touching on the impacts of climate change on the mental health of Circumpolar Indigenous Peoples. Considering most research in the field has been qualitative, we opted for a qualitative and narrative synthesis of the literature. Given the exploratory nature of this project, we applied a thematic analysis to the reviewed studies, using primarily an inductive approach to derive and interpret the themes within the scope of the research aims (Braun & Clarke, 2006; Walker et al., 2019; Williams et al., 2019).

### Search strategy

Seven databases were interrogated between July 14 and July 29, 2019: PubMed, PsycInfo, SI Web of Science, Embase, Cochrane, GeoBase, and CINAHL (see Supplementary file for review protocol and search queries). Search queries included a combination of keywords related to populations (e.g., Inuit, Saami, Indigenous), territories (e.g., Canada, Sweden, Arctic), mental health (e.g., stress, resilience, suicide), and climate (e.g., climate change, flood, global warming). Preferred Reporting Items for Systematic Review (PRISMA) guidelines and checklist were followed. A library scientist at Université Laval assisted in elaborating the search strategy. The review was updated on May 18, 2021 to include articles published up to December 31, 2020.

### Eligibility criteria

Original peer-reviewed qualitative, quantitative, and mixed-methods studies were included if: (1) they addressed emotional or mental wellbeing, psychological resilience, mental illness, or psychological problems, (2) they addressed climate change in general, or specific environmental changes related to climate change (e.g., water resources, floods, extreme weather, sea levels), and (3) the population studied was one of the Circumpolar Peoples (defined in the introduction). Language was limited to English or French, and there was no limit for the date of publication.

### Study selection

Entries were exported to Endnote. Two authors (LL, VP) independently screened the titles and abstracts of retrieved publications and excluded those that did not meet the eligibility criteria. Next, the full-text assessment of articles was conducted by LL and VP independently, and more publications were excluded for not meeting eligibility criteria. Cohen's Kappa coefficient was calculated at 0.80, which corresponds to strong interrater reliability (McHugh, 2012). Disagreement was resolved by consensus between the two reviewers.

### Data extraction and thematic analysis

The community location, sample size, characteristics of participants, study design, and study objectives (as stated in the introduction) were extracted from retained articles. Reports of climate change-related environmental transformations were also extracted from the articles. An inductive approach using thematic analysis was conducted to identify mental health impacts (Braun & Clarke, 2006; Walker et al., 2019; Williams et al., 2019). The analysis was conducted by LL and revised by VP, following the procedure developed by Braun and Clarke (2006). Direct verbatims, when available, along with narrated and synthesized results (qualitative or quantitative), were coded and grouped into themes. Coding was performed manually using a standard word-processing software. Themes were iteratively adjusted and reorganized to consistently group data across studies. To identify differences and similarities in results across sample characteristics and locations, demographic information was noted for each code and study. Lastly, all coauthors collaborated in defining, renaming, and hierarchically organizing the themes to produce a coherent synthesis.

## Results

### Study selection

**Figure 1.**
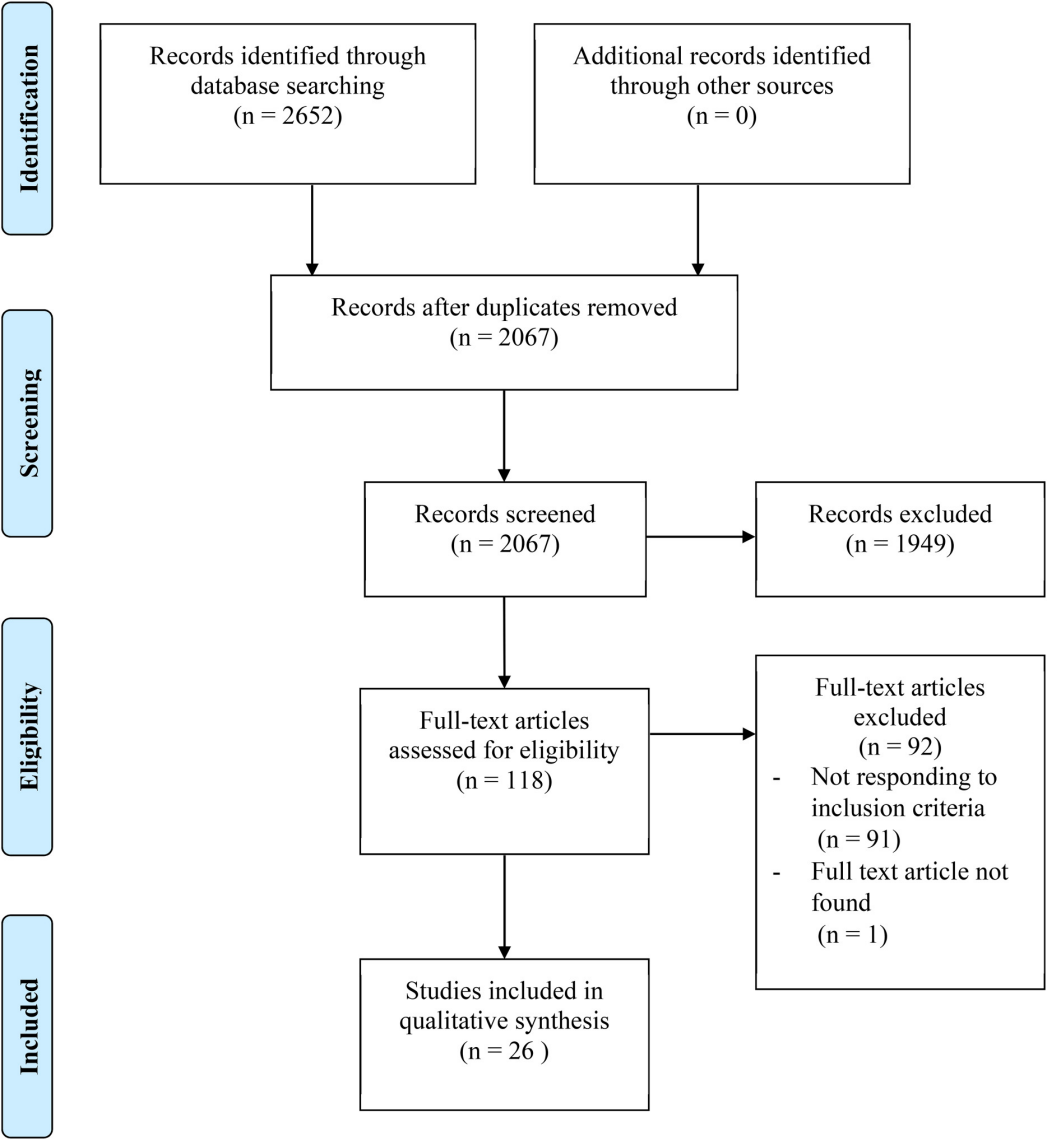
Flow chart of the systematic review.

In total, 2,652 records were identified in the database search and 26 articles met eligibility criteria after removal of duplicates (Figure 1, Table 1). Most articles included qualitative data (*n* = 25). Five also presented quantitative data (reported narratively) (Allen, 2020; Cunsolo Willox et al., 2012; Gilbert et al., 2021; Ostapchuk et al., 2012; Proverbs et al., 2020). Ten geographical regions were represented: most studies focused on Inuit communities of Nunatsiavut (*n* = 12), Nunavut (*n* = 6), Nunavik (*n* = 1), and NunatuKavut (*n* = 1), while others focused on the Dene, Cree, and Gwich’in in the Northwest Territories (*n* = 2) and in Yukon and Northern Alberta (*n* = 1), the Saami in Sweden (*n* = 1), the Inupiat and Aleut in Alaska (*n* = 5), and diverse Indigenous Peoples in the Sakha Republic in Russia (*n* = 1). Some studies included more than one region (Boulanger-Lapointe et al., 2019; MacKay et al., 2020). Several Circumpolar Peoples were not represented (e.g., Yupik, Chugach).

**Table 1.** Studies included in the systematic review and their characteristics.

Title First author (year)	Community	Sample size and characteristics of participants	Study design	Objectives
A necessary voice: Climate change and lived experiences of youth in Rigolet Petrasek MacDonald (2013)^a^	Rigolet, Nunatsiavut, Canada	*n* = 20 Age 12–25 years old	Semi-structured interviews Digital storytelling Data analysis by immersive constant comparative method Community participation in research process	To explore the observations and perceptions of climate change held by youth in Rigolet
Climate change and health effects in Northwest Alaska Brubaker (2011)	Point Hope and Kilavina Alsaka, USA	*n* = NA Alaska Natives (Inupiat) in Northwest Arctic	Case study Participatory health impact assessment	To record local observations, vulnerability factors, and responses to climate change in Point Hope and Kilavina
Climate change and mental health: An exploratory case study in Rigolet Cunsolo Willox (2013a)	Rigolet, Nunatsiavut, Canada	*n* = 67 (*n* females = 40) All ages (9–80 years old) Primarily from Rigolet (*n* = 61). Incl. government & health workers (*n* = 6)	Semi-structured, open-ended question interviews Community participation in research process	To explore the impacts of climate change on mental health and wellbeing in an Inuit context
Climate change influences on environment as a determinant of Indigenous health: Relationships to place, sea ice, and health in an Inuit community Durkalec (2015)	Nain, Nunatsiavut, Canada	Focus groups: *n* = 9 Interviews: *n* = 22 Nain residents for 20 years or more and/or frequent sea ice users and experts on the local environment as recognized by others	Sequential mixed methods (focus groups, interviews, participant observation) Data analysis by thematic analysis Community participation in research process	To investigate the relationship between sea ice and diverse aspects of health in Nain
Climate-sensitive health priorities in Nunatsiavut, Canada Harper (2015)^b^	Nunatsiavut, Canada	Photovoice workshops: *n* = 11 Community survey: *n* = 187 A member from every household in the community during the sampling period Interviews: *n* = 11 Regional health representatives	Mixed methods (semi-structured interviews, 3 photovoice workshops, 2 community surveys) Community participation in research process	To understand climate-sensitive health outcomes currently affecting Inuit in the Nunatsiavut region; to identify climate-sensitive health issues that are anticipated to affect communities in the future; and to prioritize climate-sensitive health issues to inform future research and policy development.
Disasters, migrations, and the unintended consequences of urbanization: What's the harm in getting out of harm's way? Wolsko (2016)	Shishmaref, Alaska, USA	*n* = NA Residents of Shishmaref	Mixed-method survey (ethnographic observation, interviews, surveys, investigation of state and federal documents)	To integrate research on disasters and climate change-induced migration with perspectives from environmental psychology and the psychology of natural disasters; to consider the potential costs of particular migration scenarios, applied to the case of Shishmaref
Exploring elders’ and seniors’ perceptions of how climate change is impacting health and wellbeing in Rigolet, Nunatsiavut Ostapchuk (2012)^a^	Rigolet, Nunatsiavut, Canada	Interviews: *n* = 22 Age 50+ years old, Rigolet Elders or senior Community survey: *n* = 75 28 participants above 50 years old	Mixed-methods approach (quantitative survey, qualitative interviews)	To examine the perceived impacts of climate and environmental changes on physical, mental, and emotional health, as observed by Elders and seniors in the Inuit community of Rigolet
Facing the limit of resilience: Perceptions of climate change among reindeer herding Saami in Sweden Furberg (2011)	Sápmi, Sweden	*n* = 14 (*n* females = 3) Age: 16–75 years old Reindeer herding Saami (active and retired) 11 villages represented from different regions	Semi-structured, open-ended question interviews	To explore the experiences and perceptions of climate change among Swedish reindeer-herding Saami
From this place and of this place: Climate change, sense of place, and health in Nunatsiavut, Canada Cunsolo Willox (2012)^a^	Nunatsiavut, Canada	Interviews: *n* = 72 (*n* females = 43) Age 9–85 years old Questionnaire: *n* = 112 (*n* females = 60) Age 15+ years old Residents of Rigolet	Interviews Community involvement in all stages of the research process	To examine the ways in which changes in local landscapes, the subsequent disruption of livelihoods and subsistence activities, and a changing sense of place and place-specific identities determine physical, mental, and emotional health and wellbeing
Protective factors for mental health and wellbeing in a changing climate: Perspectives from Inuit youth in Nunatsiavut, Labrador Petrasek MacDonald (2015)	Nunatsiavut, Canada	*n* = 17 Age 15–25 years old. From 5 Inuit communities in Nunatsiavut	Interviews Analysis by constant comparative method	To identify and characterize youth-specific protective factors that enhance wellbeing in light of a rapidly changing climate, and examine how climatic and environmental change challenges these
Vulnerability and adaptive capacity of Inuit women to climate change: A case study from Iqaluit, Nunavut Bunce (2016)	Iqaluit, Nunavut, Canada	Interviews: *n* = 42. Inuit women who had lived in Iqaluit for at least 5 years and who had a hunter in their family Focus groups: *n* = 13. Government, northern science organization, and Indigenous organization	Mixed method study (semi-structured interviews / focus groups, participant observation / personal conversation) Thematic analysis	To document observed changes in climate, the environment, livelihoods, and culture; to examine implications of these changes on community life and wellbeing, and identify factors resulting in differential impact; to identify strategies and coping mechanisms used to plan for, adapt to, and manage these changes (adaptive capacity); and to identify potential future impacts of climate change
Kiavallakkikput agviq (into the whaling cycle): Cetaceousness and climate change among the Inupiat of Arctic Alaska Sakakibara (2010)	North Slope Borough, Alaska, USA	*n* = 92 Age: “various generations"	Mixed method study (individual and group “ethnographic” interviews, with questionnaires and informal discussions)	To investigate how Iñupiat maintain their physical and spiritual links with the bowhead whales in ways that sustain their cultural identity and help them cope with environmental change
The land enriches the soul: On climatic and environmental change, affect, and emotional health and wellbeing in Rigolet, Nunatsiavut, Canada Cunsolo Willox (2013b)^a^	Rigolet, Nunatsiavut, Canada	*n* = 57 (*n* females = 33) Age: 9–90 years old Recommended from community research assistants and Town Council members based on regular participation in hunting, trapping, fishing, and/or travelling to cabin	Interviews Digital storytelling Community-led participatory project. Community participation throughout all stages of the research	To examine the impacts of climate change on health and wellbeing within an Inuit context, with health conceptualized as encompassing physical, mental, emotional, and spiritual processes and components
Youth engagement in climate change action: Case study on indigenous youth at COP24 MacKay (2020)	Northwest Territories, Yukon, and Alberta, Canada	*n* = 14 Youths participating in a climate conference, coordinators, chaperones, and other community members connected to the youths	Semi-directed interviews Conventional content analysis approach	To explore the value of Indigenous youth engagement in climate governance as an opportunity to offset the anxieties related to climate change
The perception of permafrost thaw in the Sakha Republic (Russia): Narratives, culture and risk in the face of climate change Doloisio (2020)	Sakha Republic (Yakutia), Russia	*n* = 24 Individuals and institutions from various occupations and fields of work	Semi-directed interviews Three interview frameworks Thematic analysis	To obtain a better understanding of the new risk patterns associated to permafrost thaw through the collection and subsequent analysis of narratives of personal experiences in order to identify the main concerns, how these are defined, and which coping strategies are considered by local inhabitants
Berry plants and berry picking in Inuit Nunangat: Traditions in a changing socio-ecological landscape Boulanger-Lapointe (2019)	Nunavut (4 villages), Nunavik (3 villages), Nunatsiavut (1 village)	*n* = 138 (*n* females = 81) Local knowledge-holders	Semi-structured interviews Complemented by archive material in Igloolik Thematic analysis	To provide a comprehensive review of the cultural and social importance of berries as well as the constraints on availability allowing for an in-depth analysis and comparison of changes in berry use through time across Inuit Nunangat
Social-ecological determinants of access to fish and well-being in four Gwich’in communities in Canada’s Northwest Territories Proverbs et al., (2020)	Northwest Territories, Canada	*n* = 29 (*n* females = 16) Individuals (*n* = 23) and groups of 2 (*n* = 3) Ages estimated between 30 and 80 years old 4 Gwich’in communities	Semi-structured interviews Thematic analysis	To examine relationships between access to fish and wellbeing; to document observations of environmental change; to explore factors preventing or helping people get fish
An update on Inuit perceptions of their changing environment, Qikiqtaaluk (Baffin Island, Nunavut) Sansoulet (2020)	Baffin Island, Nunavut, Canada	*n* = 23 (*n* females = 1) Hunters, fishers, and a project coordinator 3 Inuit villages	Semi-structured interviews 4-day land-based activity with 13 Inuit	To understand how the impacts of climate change are perceived locally in the Arctic, in the communities of Kanngiqtugaapik, Pangniqtuuq, and Qikiqtarjuaq
You can never replace the caribou: Inuit experiences of ecological grief from caribou declines Cunsolo (2020)	Nunatsiavut and NunatuKavut, Canada	*n* = 105 Community members from Nunastiavut (*n* = 75) and Nunatukavut (*n* = 30)	Conversational interviews Hybrid thematic analysis (deductive and inductive)	To characterize the lived experiences of grief and loss experienced by Inuit in Nunatsiavut and NunatuKavut, in response to the rapid decline of caribou and resulting lack of access to caribou for harvesting, and the subsequent impacts on food systems, cultural continuity, community connections, and health and wellbeing
We’re people of the snow: Weather, climate change, and Inuit mental wellness Middleton (2020a)	Nunatsiavut, Canada	*n* = 116 (*n* females = 52) Community members (*n* = 96) Health professionals (*n* = 20)	Semi-structured interviews Hybrid thematic analysis (deductive and inductive)	To characterize: (1) the personal and collective significance of climate, including changing weather and seasonal patterns, among Nunatsiavut Inuit; and (2) how weather, season, and climate influence mental wellness in the context of climate change
Sharing country food: Connecting health, food security and cultural continuity in Chesterfield Inlet, Nunavut Newell (2020)	Chesterfield Inlet, Nunavut, Canada	*n* = 36 Elders, youth, and hunters included	Interviews (*n* = 27) Open-house meetings (*n* = 9) Thematic analysis Community-based research	To develop a theoretical framework of how food security, cultural continuity, and community health and wellbeing are interconnected to allow for a richer understanding of how increased shipping, climate change, and social changes are impacting community members
“The best scientists are the people that's out there”: Inuit-led integrated environment and health monitoring to respond to climate change in the Circumpolar North Sawatzky (2020)	Rigolet, Nunatsiavut, Canada	*n* = 31 Community members (*n* = 13) Inuit government representative (*n* = 14) Inuit health professionals (*n* = 4)	Semi-structured interviews Community participation Hybrid thematic analysis (deductive and inductive)	To identify and characterize what an integrated monitoring system should include if it is to be Inuit-led, and Inuit-focused, based on the perspectives, knowledge, and lived experiences of Inuit living in Rigolet, Canada
Determinants, effects, and coping strategies for low-yield periods of harvest: A qualitative study in two communities in Nunavut, Canada Gilbert (2021)	Cambridge Bay and Pond Inlet, Nunavut, Canada	*n* = 30 Elders and/or harvesters	Semi-structured interviews Validation meetings Thematic analysis	To examine the meaning of country food, to identify determinants of low-yield periods of country food harvest and their effects on community health, and to summarize coping strategies and ideas for sustaining food security during these “leaner” periods
“What are you going to do, protest the wind?”: Community perceptions of emergent and worsening coastal erosion from the remote Bering Sea Community of St. Paul, Alaska Tran (2021)	St. Paul, Alaska, USA	*n* = 21 (*n* females = 10) St. Paul residents Age 8 years old to late 70s Various backgrounds	Interviews Focus groups Inductive coding analysis	To inform St. Paul's erosion monitoring and climate adaptation strategies by documenting community perceptions of coastal erosion as an ecological and social threat within a broader context of multiple established climate stressors
Being on land and sea in troubled times: Climate change and food sovereignty in Nunavut Panikkar (2020)	Cambridge Bay and Kugluktuk, Nunavut, Canada,	*n* = 25 (*n* females = 4) Elders and experienced hunters	Semi-structured interviews Coding analysis	To understand impacts from climate change on food sovereignty and health of people in Kugluktuk and Cambridge Bay
Climate change in Alaska: Social workers’ attitudes, beliefs, and experiences Allen (2020)	Alaska, USA	*n* = 159 Social workers, including Alaska Natives (*n* = 18)	Web-based survey 89 closed-ended questions	To assess social workers’ attitudes to climate change and their perceptions of the effects of climate change on the individuals, families, organizations, and communities with whom they work in Alaska

NA = not available.

^a^ Changing Climate, Changing Health, Changing Stories project.

^b^ Indigenous Health Adaptation to Climate Change project.

### Theme 1: The land, the self, and climate change

#### Importance of the land for mental health

The natural environment was found to foster a sense of true self and to be a core component of Inuit, Inupiat, and Gwich’in identities (Bunce et al., 2016; Cunsolo et al., 2020; Cunsolo Willox et al., 2012, 2013a, 2013b; Harper et al., 2015; Middleton et al., 2020a; Ostapchuk et al., 2012; Petrasek MacDonald et al., 2015; Proverbs et al., 2020; Sakakibara, 2010; Sawatzky et al., 2020; Wolsko & Marino, 2016). In three studies, Inuit reported that the land connects them with their ancestors and with nature, and that it allows spiritual enrichment, continuity of traditions, and remembrance of history (Cunsolo Willox et al., 2013b; Durkalec et al., 2015; Petrasek MacDonald et al., 2015). In nearly half of the articles, land-based activities, which are taught across generations, were reported to be essential for cultural pride, self-value, confidence, motivation, and a sense of self-determination among Inuit and Gwich’in (Bunce et al., 2016; Cunsolo et al., 2020; Cunsolo Willox et al., 2012, 2013a, 2013b; Durkalec et al., 2015; Harper et al., 2015; Middleton et al., 2020a; Petrasek MacDonald et al., 2015; Proverbs et al., 2020). Sea ice- and land-based cultural activities were felt to provide a sense of freedom of movement and autonomy, which was linked to mental, emotional, spiritual, social, and cultural wellbeing, as well as to the Inuit identity (Durkalec et al., 2015; Middleton et al., 2020a; Ostapchuk et al., 2012). Several interviewees in Nain, Nunatsiavut, expressed that, traditionally and still today, they feel less within reach of colonial institutions, policies, and racism when on the land (Durkalec et al., 2015).

Inuit and Inupiat individuals from 11 studies perceived that the integrity of their connection to the land is essential for their mental health and resilience (Boulanger-Lapointe et al., 2019; Cunsolo Willox et al., 2012, 2013a, 2013b; Harper et al., 2015; Ostapchuk et al., 2012; Petrasek MacDonald et al., 2013, 2015; Sansoulet et al., 2020; Sawatzky et al., 2020; Wolsko & Marino, 2016). Eight of the studies found that for many Inuit, going outside the community (i.e., travelling or performing activities on the land) is an important way of dealing with the stress and problems of daily life (Boulanger-Lapointe et al., 2019; Bunce et al., 2016; Cunsolo Willox et al., 2012, 2013a, 2013b; Durkalec et al., 2015; Panikkar & Lemmond, 2020; Petrasek MacDonald et al., 2015). In Nunatsiavut, Nunavik, and Nunavut, Inuit participants of 10 studies reported feeling more relaxed, calm, and peaceful, as well as healthier and happier, when on the land (Boulanger-Lapointe et al., 2019; Bunce et al., 2016; Cunsolo Willox et al., 2012, 2013a, 2013b; Durkalec et al., 2015; Harper et al., 2015; Middleton et al., 2020a; Petrasek MacDonald et al., 2015; Sawatzky et al., 2020). An Inuit youth also explained that being on the land fosters better habits such as decreased substance misuse (Petrasek MacDonald et al., 2015). Engagement in land-based activities, such as hunting or fishing, was identified as directly contributing to physical and mental wellbeing in 11 studies in Nunatsiavut, Nunavut, and the Northwest Territories (Cunsolo Willox et al., 2012, 2013a, 2013b; Durkalec et al., 2015; Gilbert et al., 2021; Harper et al., 2015; Newell et al., 2020; Panikkar & Lemmond, 2020; Petrasek MacDonald et al., 2013; Proverbs et al., 2020; Sansoulet et al., 2020). Particularly in studies among Inuit populations in Canada, the land is identified to be intimately linked to identity and wellbeing through local traditional foods, also known as country foods, which have strong cultural significance and are important for food security as well (see Theme 2 below) (Cunsolo et al., 2020; Gilbert et al., 2021; Newell et al., 2020; Panikkar & Lemmond, 2020; Sansoulet et al., 2020). Country foods are traditional foods that are hunted, fished, or harvested on the land or in the rivers or the sea, such as caribou, seal, beluga, narwhal, Arctic char, geese, molluscs, or berries.

#### Climate change disrupts relationships with the land

Across the Circumpolar North, climate change disrupts physical access to the land (Table 2), thereby threatening peoples’ connection to the land, and impacting multiple determinants of their mental health and resilience. For instance, because of unpredictable weather, storms, and fragilized ice, accidents are increasingly frequent in Alaska, Nunavut, and Nunatsiavut, causing non-intentional injuries and sometimes death (Brubaker et al., 2011; Bunce et al., 2016; Durkalec et al., 2015; Panikkar & Lemmond, 2020; Sakakibara, 2010; Sansoulet et al., 2020; Wolsko & Marino, 2016). Hence, for some, climate change was associated with the loss of loved ones (Durkalec et al., 2015). Meanwhile, Middleton et al. (2020a) found that transitions between seasons were periods of agitation and distress among Inuit communities of Nunatsiavut, and that climate change exacerbated this stress by lengthening seasonal transitions. As well, hostile environment and poorer travelling conditions may trigger individual-, family-, and community-level anxiety or concerns about the limited access to the land and its safety hazards; this was reported in Nunavut, Nunatsiavut, Alaska, and the Sakha Republic (Bunce et al., 2016; Doloisio & Vanderlinden, 2020; Durkalec et al., 2015; Harper et al., 2015; Middleton et al., 2020a; Newell et al., 2020; Panikkar & Lemmond, 2020; Petrasek MacDonald et al., 2013; Sakakibara, 2010; Sansoulet et al., 2020; Wolsko & Marino, 2016). One study also found that sometimes, in Inuit communities, weather forecasts themselves triggered anxiety related to the uncertainty of being able to go on the land or not (Middleton et al., 2020).

**Table 2.** Environmental changes reported across regions of the Circumpolar North.

Region of the Circumpolar North	Environmental changes reported
Nunatsiavut and NunatuKavut (Canada)	Seasonal changes: warmer temperatures on average, hotter summers and milder winters, unusual seasonal timing, increased exposure to thermal extremes. Increase of incidents related to freezing-thawing-freezing cycles Changes in storms: timing, increased intensity, frequency, and duration Changes in precipitation (quality, quantity, stability, formation, extent): *Rain*: increased frequency and intensity of rainfall *Snow*: earlier accumulation and melting; different consistency Changes in the ice: decrease in ice quality and thickness of sea ice. Ice melting later in the year Changes in vegetation: new plants, others disappearing. Decrease in quality and taste of berry plants / better berry quality Changes in wildlife: migration patterns of some animals changing, new animals, increase in flies and mosquitoes, decreased quality of wild meat, change in taste, changes in animal fur thickness and quality, decline of some species (e.g., caribou) Changes in water: ponds and lakes drying up, changes in coastline water heights, decrease in freshwater quality and abundance
Nunavut (Canada)	Changes in the ice: thinner and softer sea ice, changed timing of freezing and breaking Changes in precipitation: snow and rain Changes in vegetation: berry quantity and quality, flowering cycles Changes in wildlife: declining animal populations (caribou, seals, musk ox, narwhal, Arctic char), changing animal pattern mammals (increased unusual presence of bears, orcas, whitefish), increasing numbers of mosquitoes. More sickness among animals Changes in weather: increased wind, storms, rising sea temperature, fog, warmer temperature
Alaska (USA)	Changes in weather: unpredictability, extreme weather, altered seasons, temperature changes, changes in snow and rain Changes in sea ice thickness Increased erosion, windiness, and flooding disaster Changing ocean currents, poorer water conditions Changes in wildlife
Northwest Territories, (Canada)	Changes in weather and landscape: temperature, seasonal changes, storm, wind, precipitation, permafrost thaw, landslides Changes in vegetation Changes in fish: health, migration, quantity, flesh Changes in rivers and lakes: morphology, current, water levels and quality, ice dynamics
Sakha Republic (Russia)	Changes in wildlife and vegetation Increasing erosion and flooding events Thawing permafrost
Sápmi (Sweden)	Changes in weather: unstable and extreme weather Seasonal changes: longer, wetter, and warmer autumn, waters freezing later in the year, no autumnal night frost, warmer winters, early and sudden spring, irregular summers Changes in vegetation: grazing land shrinking, mushrooms not freezing, tree line getting in the north Changes in wildlife: migratory patterns of animals
Nunavik	NA
Yukon	NA
Northern Alberta	NA

NA = not available.

The rapidity of environmental changes challenges the adaptation of local cultures and knowledge (Brubaker et al., 2011; Cunsolo et al., 2020; Sansoulet et al., 2020). Fundamental skills are needed to properly hunt, fish, harvest, and prepare country foods, as well as to sew animal skin and travel on the land. But the opportunities to maintain, adjust, or acquire these practices are decreasing (Cunsolo et al., 2020; Gilbert et al., 2021; Harper et al., 2015; Panikkar & Lemmond, 2020; Petrasek MacDonald et al., 2013, 2015; Sansoulet et al., 2020). Interviewees from two studies felt that trusted Inuit knowledge about travelling conditions and land-based activities has become less valid under the changing climate (Durkalec et al., 2015; Harper et al., 2015). Two studies found that this mismatch limits individuals’ capacities to interact with the land and to deepen their relationship with it (Harper et al., 2015; Sawatzky et al., 2020).

[When] you’re growing up, you’re taught where to go and where not to go ‘cause there's bad parts of the ice. Ah, it seems like today or this past winter, you had [bad] places that are existing where they shouldn't be, where they weren't before. (Harper et al. 2015, p. 13).

As such, participants believed “that knowledge is still passed on; [but] the relevance of it all becomes a question” (community member of Rigolet) (Harper et al., 2015). Hence, there are fears and worries that cultural land-based skills and knowledge could be lost (Cunsolo et al., 2020; Harper et al., 2015; Petrasek MacDonald et al., 2013, 2015; Sansoulet et al., 2020). Sadness, anxiety, and frustration about this situation have been reported among Inuit in four articles (Cunsolo et al., 2020; Gilbert et al., 2021; Petrasek MacDonald et al., 2013, 2015).

Climate change-related barriers to reindeer herding in Sweden illustrate the impacts on mental health of these cultural and social disruptions. Reindeer herding holds a central place in the economy and identity of the Saami people. Climate change affects the adequacy of traditional herding methods and the feasibility of reindeer herding, a sector already pressured by industrialization (Furberg et al., 2011). Consequently, in the study by Furberg et al. (2011), Saami participants reported stress about traditional herding, and grief for its future. This grief can also be found in Inuit communities of Nunatsiavut, where caribou are in decline and cannot be hunted anymore (Cunsolo et al., 2020). Some Saami interviewees also mentioned that climate change-related predictions constituted an even greater source of stress than the current changes themselves, in the same way that anticipated changes in some Inuit communities were related to mental health difficulties (Middleton et al., 2020a). The situation also affects the people's relationship with researchers and authorities. Saami participants highlighted an underrepresentation of their people and of reindeer herding in research and reports, leading them to feeling unrecognized by Swedish society (Furberg et al., 2011). They reported injustice from a lack of institutional support from authorities to deal with environmental changes they have not caused. Feelings of resignation and powerlessness were reported amongst this cultural and economic upheaval. These feelings were reinforced by their evoking of traumatic memories of the 1986 Chernobyl accident, where the Saami were similarly affected by another form of environmental upheaval beyond their control (Furberg et al., 2011).

As climate change transforms the environment and disrupts livelihoods, worries about loss of culture and autonomy have been observed in nine studies across the Circumpolar North (Cunsolo et al., 2020; Cunsolo Willox et al., 2012; Durkalec et al., 2015; Furberg et al., 2011; Harper et al., 2015; Ostapchuk et al., 2012; Panikkar & Lemmond, 2020; Petrasek MacDonald et al., 2013, 2015). In Inuit communities, the sense of self-worth and purpose is affected when people are unable to perform land-based activities and feed their families (Bunce et al., 2016; Cunsolo et al., 2020; Cunsolo Willox et al., 2013a; Harper et al., 2015). An experienced hunter and trapper in Rigolet explained that not being able to go out on the land feels “like you got some kind of handicap” (Cunsolo Willox et al., 2013a, p. 20). Some even perceived a loss of their cultural identities (Cunsolo et al., 2020; Cunsolo Willox et al., 2013a; Petrasek MacDonald et al., 2013), with disconnection from the natural environment being felt to limit one's true self (Bunce et al., 2016; Cunsolo Willox et al., 2013a, 2013b; Sawatzky et al., 2020). In the Sakha Republic and in Alaska, various erosion processes attributed to climate change were found to impact culture and wellbeing by deteriorating cultural places such as cemeteries, disrupting place attachment among residents, and altering their close emotional ties to the land (Doloisio & Vanderlinden, 2020; Tran et al., 2021).

#### Associations with colonization and past traumas

These imposed constraints on core components of Circumpolar lifestyles and identities were felt to magnify the traumas of colonization in two studies (Cunsolo Willox et al., 2013a; Harper et al., 2015). Because they are not able to go on the land, interviewees in Rigolet reported feeling trapped in negative thoughts related to past traumas: “I think that those effects [of trauma from residential schools and assimilation] will be felt further if climate change affects [land] activity” (Rigolet community member) (Cunsolo Willox et al., 2013b, p. 261). There was also a perceived decrease of resilience to climate change because of previous traumas, leading to greater vulnerability to climate-related mental health issues (Cunsolo Willox et al., 2013a). Similarly, one study found that many interviewees in Gwich’in communities identified barriers to fish access stemming from both climate change and colonization, in turn impacting their wellbeing (Proverbs, 2020).

### Theme 2: Social and dietary dimensions of climate change

#### Effects of climate change on social spaces

One of the main benefits for mental health associated with land-based activities is their creating a space for positive interpersonal relationships (Cunsolo et al., 2020; Durkalec et al., 2015; Harper et al., 2015; Petrasek MacDonald et al., 2013, 2015). As an example, Inuit in Nunatsiavut and NunatuKavut explained how hunting caribou linked together families, communities, the land, and the food (Cunsolo et al., 2020). Because climate change constrains travel opportunities, almost half of the studies found that Inuit and Inupiat are confined within their communities for increasingly longer periods of time during the year, leading to feeling trapped, and sometimes to stress and anxiety (Brubaker et al., 2011; Bunce et al., 2016; Cunsolo Willox et al., 2013a, 2013b; Durkalec et al., 2015; Ostapchuk et al., 2012; Panikkar & Lemmond, 2020; Petrasek MacDonald et al., 2013; Sakakibara, 2010; Wolsko & Marino, 2016). Women in Nunavut observed more stress in their families and households as a consequence of longer time spent in confined spaces (Bunce et al., 2016). Increased intrafamilial tension was reported in Nunavut and Nunatsiavut (Bunce et al., 2016; Cunsolo Willox et al., 2013a; Petrasek MacDonald et al., 2013, 2015). More generally, four studies found that less time on the land, a safe place for social and intergenerational connections, was also linked to negative impacts on relationships with others (Cunsolo Willox et al., 2012; Harper et al., 2015; Petrasek MacDonald et al., 2013, 2015). In Nunatsiavut, decreased possibilities to go on the land may aggravate existing overcrowding-related mental health problems (Cunsolo Willox et al., 2013a; Harper et al., 2015). Further, worries about the changing ice and its risks were said to pervade everyday social interactions (Cunsolo Willox et al., 2013b). For Nunatsiavut youth, land-based activities were found to foster supportive social networks, good role models, trust, and solidarity. Barriers to travel were felt to jeopardize the essential role of land-based activities in facilitating these interactions (Petrasek MacDonald et al., 2015).

#### Effects of climate change on traditional diets

Access to traditional or country food, the ability to share it with others, and the associated family and community time were reported to foster individual wellbeing, community strength, and food security for Inuit in Nunavut and Nunatsiavut, as well as for Gwich’in in the Northwest Territories (Cunsolo et al., 2020; Gilbert et al., 2021; Newell et al., 2020; Panikkar & Lemmond, 2020; Proverbs et al., 2020). Several studies mentioned that these highly valued country foods are becoming increasingly scarce because of disruptions in marine and terrestrial ecosystems (Table 2) (Cunsolo Willox et al., 2012; Furberg et al., 2011; Gilbert et al., 2021; Panikkar & Lemmond, 2020; Proverbs et al., 2020; Sakakibara, 2010). For example, Sakakibara (2010) reported that in some coastal Inupiat communities, hunting bowhead whales is no longer possible during certain periods of the year due to new migratory patterns. A few kilometers to the south, other communities did not face this issue, illustrating how much such impacts can be location specific. Deep emotional reactions such as discouragement and loss, among others, were linked to constrained access to country food (Cunsolo et al., 2020; Gilbert et al., 2021; Panikkar & Lemmond, 2020). Declining hunting skills and impacts on self-confidence were also identified as consequences (Panikkar & Lemmond, 2020). One study in Nunavut found that Elders, children, young adults, individuals with low income, and individuals with physical illnesses were particularly affected by reduced access to country food (Gilbert et al., 2021). Disconnection from culture was also a concern in Kugluktuk (Nunavut), where people started to sell country food instead of sharing it because of economic necessity (Panikkar & Lemmond, 2020).

Overall, these new barriers to country food aggravate pre-existing food insecurity in the North (Berner et al., 2016; Expert Panel on the State of Knowledge of Food Security in Northern Canada, 2014; Newell et al., 2020; Sansoulet et al., 2020; Walch et al., 2018). Seeing many turning to store-bought and less healthy foods, community members in five studies have reported anxiety, concerns about the future, and a feeling of disconnection from their Inuit identities, food preferences, and practices (Bunce et al., 2016; Cunsolo et al., 2020; Ostapchuk et al., 2012; Petrasek MacDonald et al., 2013; Wolsko & Marino, 2016).

#### Impacts specific to social roles

Different social roles may underlie distinct experiences of climate change in men and women. In Iqaluit, Nunavut, women explained how rapid socio-environmental changes could affect their social roles by preventing them from feeding theirs families and performing traditional activities, like cleaning and sewing animal skins (Bunce et al., 2016). In an Inuit community of Nunatsiavut, men reported lack of confidence and nervousness about climate-related changes, whereas women reported loss of motivation and disappointment from travel limitations (Durkalec et al., 2015). Also in Nunatsiavut, constraints in young men's opportunities and knowledge transmission for hunting may deprive them from feeling the pride and sense of self-worth associated with this activity (Cunsolo et al., 2020).

Finally, experiences from Nunatsiavut suggest that environmental changes may impact mental health services by increasing the demand, by preventing health workers from getting out on the land in their leisure time, by increasing the frequency of communication outages due to extreme weather, and by compromising land-based mental health programs (Cunsolo Willox et al., 2013a; Harper et al., 2015; Middleton et al., 2020a).

### Theme 3: Mental health outcomes

#### Emotions and behaviors

Together, these multidimensional effects of climate change, mediated by cultural, social, economic, physical, and psychological determinants of wellness, were found to translate into several affective reactions and negative behaviors in most studies. Depressive reactions, such as grief, sadness, boredom, suicidal ideations, and self-pity, were reported in Nunatsiavut and Nunavut (Bunce et al., 2016; Cunsolo et al., 2020; Cunsolo Willox et al., 2013b; Gilbert et al., 2021; Middleton et al., 2020a). Anxiety, fear, frustration, and anger were reported in Nunatsiavut communities (Cunsolo et al., 2020; Cunsolo Willox et al., 2013b; Middleton et al., 2020a; Ostapchuk et al., 2012; Petrasek MacDonald et al., 2015), in the Sakha Republic (Doloisio & Vanderlinden, 2020), in Nunavut (Gilbert et al., 2021; Panikkar & Lemmond, 2020), in Alaska (Tran et al., 2021), and among youth in the Mackenzie River basin in Canada (MacKay et al., 2020). Many found that boredom, isolation, and feeling trapped are contributing to substance misuse, spousal abuse, and suicide (Cunsolo Willox et al., 2012, 2013a; Middleton et al., 2020a, 2020b; Ostapchuk et al., 2012). Behaviors such as violence and gambling were reported by some participants as possible consequences of, and responses to, environmental disruptions (Bunce et al., 2016; Cunsolo Willox et al., 2013a). Some social workers in Alaska similarly noted, in local Indigenous communities, anxious reactions, cultural loss, suicidal ideation, and increased risk for alcohol and drug misuse that they linked to climate change (Allen, 2020).

#### Appraisals and apprehensions

Individual- and community-level anxiety about the cultural future of youth was endorsed by Inuit and Saami (Cunsolo Willox et al., 2012; Furberg et al., 2011; Ostapchuk et al., 2012). Four studies found that participants were concerned about the ability to adapt while keeping a traditional lifestyle (Harper et al., 2015; Ostapchuk et al., 2012; Panikkar & Lemmond, 2020; Petrasek MacDonald et al., 2015), and that if climate change leads to the disappearance of ice and ice roads, their transportation and health would be negatively affected (Durkalec et al., 2015). In coastal communities of Alaska, the increased frequency of natural disasters, such as storms and flooding, was reported to provoke community-wide anxiety and concerns about adaptation (Brubaker et al., 2011; Sakakibara, 2010; Wolsko & Marino, 2016). For example, the inhabitants of Shishmaref reported continuously apprehending the threat of urban relocation in the event of destructive floods (Wolsko & Marino, 2016). Because their subsistence depends on the land and ocean, residents fear the impacts of relocation on food security and cultural vitality and identity. Further, they report a lack of financial support from governmental institutions to allow such transition. As a result, the threat of relocation is felt to be more distressing than the immediate risk of flooding. To illustrate this chronic stress among Shishmaref residents, one mentioned in an interview that worrying about storms “[is] what gives [him] grey hairs!” (Wolsko & Marino, 2016, p. 419). Feelings of lack of institutional support and of tangible actions to address environmental changes and imminent displacement were echoed in other communities of Alaska (Doloisio & Vanderlinden, 2020), and in the Sakha Republic (Tran et al., 2021). In both regions, lived or anticipated environmental migrations were also linked to disruptions in culture- and place-based attachment.

Within communities and across the Circumpolar North, divergent hopes, fears, and perceived capacities to adapt are reported. In Sápmi, Sweden, some parents try to remain optimistic about the uncertainty of their children's future, while others think there may be a disconnection between the hopes and reality of the children who want to continue to herd reindeers (Furberg et al., 2011). In Nunatsiavut, many Elders are concerned about the younger generation and their potential loss of culture (Cunsolo et al., 2020; Panikkar & Lemmond, 2020; Petrasek MacDonald et al., 2013). Some Elders are not hopeful they can restore traditional livelihoods before the new generation grows up, becomes further disconnected from their environment, and loses interest (Cunsolo et al., 2020). Youth from Nunatsiavut expressed fear about life-changing circumstances, and concerns about how community members were using negative ways of coping. Conversely, some people try to avoid thinking about the worst-case scenarios and hope for the best (Cunsolo Willox et al., 2013b), while some youths report being confident to be able to deal with those changes even if they worsen with time (Petrasek MacDonald et al., 2015). Various concerns about the land, its components, and their future were shared in Gwich’in communities (Proverbs et al., 2020).

### Theme 4: Responding to climate change

Across the Circumpolar North, several responses to climate change were identified. Saami interviewees in Sweden felt there is an opportunity to adapt to climate change, and that there might also be “positive” environmental changes in some areas (Furberg et al., 2011). In Inuit communities, there were mixed perceptions of positive versus negative impacts of climate change on berry quality (Boulanger-Lapointe et al., 2019; Bunce et al., 2016). Inuit communities in Nunatsiavut and Nunavut reported adaptive or resilient responses, such as learning traditional crafts and skills, doing sports, focusing on work, spending more time with family and friends, participating in music activities, building cabins closer to have an easier access, going to a youth center, and taking walks around town (Bunce et al., 2016; Cunsolo Willox et al., 2013a, 2013b; Ostapchuk et al., 2012; Petrasek MacDonald et al., 2013, 2015). In some Inuit communities, an increased interest of youth to learn their own culture was identified (Petrasek MacDonald et al., 2013). Similarly, in the Mackenzie River basin, a number of youths are positively engaged in climate change action and expressed interest in rediscovering the land (MacKay et al., 2020). Finally, in response to harsh and unpredictable environmental conditions, Inupiat communities have reinforced cultural and community bonds by passing on stories and other cultural elements, and by sharing food with other villages that were in more precarious positions due to climate change (Sakakibara, 2010).

Initiatives to monitor climate change through an Indigenous lens were considered as a way to reinforce adaptation to environmental changes (Sawatzky et al., 2020; Tran et al., 2021). One study found that many community members in Nunatsiavut felt their awareness of and response to these changes empowered them to give back to the land, in turn helping them keep and reinforce their cultural connection and attachment to the land (Sawatzky et al., 2020). Unangan (Aleut) and Gwich’in also endorsed a renewed sense of the importance of the land, and a feeling of empowerment from participating in climate monitoring (Proverbs et al., 2020; Tran et al., 2021). Of note, however, the impacts of climate change sometimes felt magnified to the people monitoring them (Sawatzky et al., 2020). Generally, Inuit in Nunatsiavut reported that sharing information about environmental changes has multiple benefits: it leads to increased awareness, facilitates adaptation of traditional knowledge, supports safety on the land, prepares people for more significant changes, and enables them to advocate for their health (Sawatzky et al., 2020). A number of youths in the Mackenzie River basin participated in the United Nations Conference of Parties on Climate Change, which gave them a voice to share their experiences, but also an opportunity to increase their own awareness of the climatic situation (MacKay et al., 2020). Through this activity, they felt a stronger connection to their land, a higher confidence and pride about their culture, and in some cases a desire to continue advocacy in their communities, further nourishing a sense of empowerment and leadership. Together, these experiences demonstrate how Indigenous-led initiatives can reinforce adaptation and wellbeing through awareness, cultural connection, and empowerment.

Various factors may participate in greater resilience to climate change. In Inupiat communities, a strong social capital (i.e., connections between individuals or communities) and cultural integrity were linked to better adaptation to environmental changes (Sakakibara, 2010; Wolsko & Marino, 2016). Youth from the Mackenzie River basin and from Nunatsiavut expressed the importance of feeling connected to others to sustain their motivation and resilience (MacKay et al., 2020; Petrasek MacDonald et al., 2015).

Socio-economics conditions were noted to influence resilience in Inuit and Inupiat (Bunce et al., 2016; Sakakibara, 2010). In particular, resources for hunting (adapted gear or financial means) were found to promote better physical and mental health, while isolation and lower socio-economic status were linked to lower adaptive capacity (Bunce et al., 2016). Generally, climate change-related barriers to land-based activities seemed easier to alleviate with financial means, whether these means were individual or collective. For example, having the possibility to organize flights to go and pick berries or to replace snowmobiles that had drowned facilitated adaptation to climate change (Bunce et al., 2016). For a group of women in Nunatsiavut, “mental health and physical wellness, a strong western and/or traditional educational foundation, money, food security, strong social networks, and a connection to Inuit identity” were identified as factors that influence their adaptive capacity and resilience to climate change (Bunce et al., 2016, p. 1432).

Ways of adapting land-based practices have been reported in Nunatsiavut, Nunavut, and Alaska, such as finding new places to hunt, pick berries, and build cabins, performing these activities at a different time of the year, participating in support programs, and reinforcing cultural activities (Bunce et al., 2016; Petrasek MacDonald et al., 2015; Sakakibara, 2010). Technologies and infrastructures significantly contributed to the adaptation of communities in Nunatsiavut and Alaska (Brubaker et al., 2011; Harper et al., 2015; Ostapchuk et al., 2012; Sakakibara, 2010; Sansoulet et al., 2020). Strategies included the use of alternative transportation means (e.g., greater use of boats) (Ostapchuk et al., 2012), beacons to facilitate search and rescue, and infrastructures protecting against extreme weather events (e.g., revetment wall for direct coastal protection) (Brubaker et al., 2011). These adaptation strategies, sometimes merged with land-based knowledge, were found to alleviate stress among individuals in Alaska (Brubaker et al., 2011). Even if land-based knowledge and practices must be adapted, people in Nunatsiavut and in Alaska see these changes as compatible with their cultures and traditions: “[It's] almost always the same message … the general part of it is still there, you just have to apply it differently now ‘cause things have changed” (Rigolet community member, Nunatsiavut) (Harper et al., 2015, p. 13); “Technology helps us carry on our relationship with the whales. It doesn't hurt the whale or our tradition” (Inupiat from North Slope Borough, Alaska) (Sakakibara, 2010, p. 1008).

## Discussion

**Figure 2.**
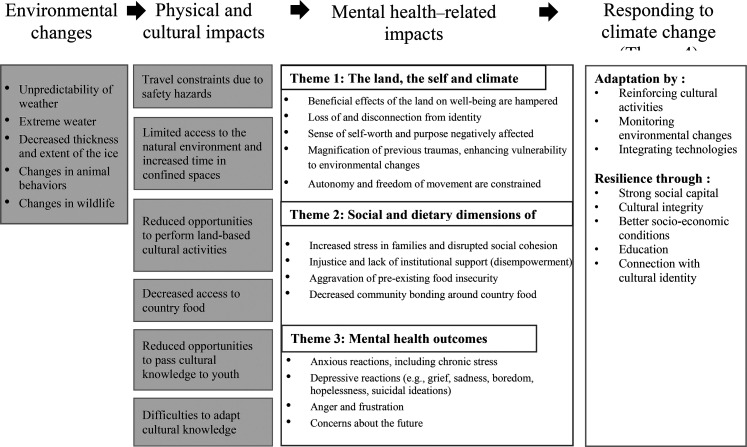
The mental health-related impacts of environmental changes in the Circumpolar North.

In this study, we systematically reviewed the literature on how climate change impacts mental health and wellness in the Circumpolar North. We found 26 original research papers exploring this question. Qualitative data from Alaska, Northern Canada, Sápmi, and the Sakha Republic illustrate how environmental changes affect mental health through multiple pathways (Figure 2).

In Sápmi, where climate change threatens reindeer herding, distress emerges as a consequence of cultural and economic insecurity, as well as grief for the future (Furberg et al., 2011). In Nunavut, Nunatsiavut, Northwest Territories, and Alaska, limited access to the land, barriers to travel, and safety hazards contribute to the fear of cultural erosion, food insecurity, and loss of social connectedness, leading to individual- and community-wide distress (e.g., Brubaker et al., 2011; Bunce et al., 2016; Cunsolo Willox et al., 2012; Proverbs et al., 2020). In the Sakha Republic, Russia, climate change led to cultural disruptions, stress, and altered identity and sense of belonging (Doloisio & Vanderlinden, 2020). These pressures are widely felt through various reactions that we broadly categorize as anxious and depressive, and they are also felt in terms of anger and loss (e.g., Durkalec et al., 2015; Furberg et al., 2011; Sakakibara, 2010). Overall, our results are congruent with the findings from Middleton et al. (2020b), whose recent literature review on worldwide Indigenous mental health and climate change outlined pathways related to place attachment, the threat of forced migration and cultural loss, and food insecurity. We build on their landmark review by zooming in on the similarities across and differences within the Circumpolar North, and by integrating the substantial new research published since their review was conducted. Half of the studies we reviewed were published in the last two years, illustrating how rapidly the field is evolving, with increasing testimonies to the mental health impacts of climate change and adaptation strategies across the Circumpolar North.

The relationship with the land plays an important role in differentiating the impacts of climate change on mental health. In the Circumpolar North, individuals with identities or social roles more closely tied to the environment were more impacted by the changes (Kelman & Næss, 2019; Middleton et al., 2020b). This resonates with literature from Australia, New Zealand, Tuvalu, and Vietnam, which highlights how climate change is more likely to affect the wellbeing of peoples whose identities, modes of subsistence and living, and knowledge systems are more closely linked with their natural environment (Cunsolo & Ellis, 2018; Gibson et al., 2019; Johnson et al., 2021; Son & Kingsbury, 2020).

We found that climate change affects mental health by constraining access to the land and by altering the land's resources. By reducing access to the land among Inuit communities, climate change deprives individuals from a central, culturally significant way of nurturing individual and community wellbeing (Boulanger-Lapointe et al., 2019; Bunce et al., 2016; Cunsolo Willox et al., 2012, 2013a, 2013b; Durkalec et al., 2015; Panikkar & Lemmond, 2020; Petrasek MacDonald et al., 2015). Similarly, in Northern Australia, mental health issues related to climate change are rooted in peoples’ connection to the land, involving feelings of loss and distress, and anxious reactions (Horton et al., 2010). Access to country foods is affected by changes in ecosystems, decline in species, and/or hunting bans, as reported in Canada and Alaska (Cunsolo et al., 2020; Cunsolo Willox et al., 2012; Furberg et al., 2011; Gilbert et al., 2021; Panikkar & Lemmond, 2020; Proverbs et al., 2020; Sakakibara, 2010). Similarly, in the Mangakāhia Valley in New Zealand, decreasing water levels and rising water temperatures have led to a decline in eel and crayfish availability, two important traditional foods for local Māori residents (Johnson et al., 2021). Among both Inuit and Māori, the reduced availability of country foods aggravates ongoing food insecurity, in addition to constraining food-sharing within communities, thereby affecting physical wellbeing, social belonging, as well as emotional and cultural connection to place. Hence, climate change affects mental health by disrupting access to the land and to country foods.

Further impacts on mental health are felt through disruptions of knowledge systems and cultural identities. Traditional knowledge of the land is no longer matching environmental reality under a changing climate, and, as a result of these changes, Circumpolar Peoples experience distressing disruptions in their livelihoods, attachment to place, and identities (Cunsolo et al., 2020; Furberg et al., 2011; Harper et al., 2015; Petrasek MacDonald et al., 2013, 2015; Sansoulet et al., 2020). Similarly, farmers from the Australian Wheatbelt, affected by the chronic dryness of their lands, have reported loss of confidence in and distress from the unpredictable weather (Cunsolo & Ellis, 2018). These environmental changes in Australia undermined their identities as farmers and stewards of the land. In rural communities of the Northern Mountainous Region (NMR) of Vietnam, some young adults have expressed more enthusiasm for new technologies rather than for traditional knowledge and skills, leading to community concerns about the cultural perennity of their local ethnic minorities (Son & Kingsbury, 2020). In the studies reviewed here, there were shared concerns about loss of culture, the identity and livelihoods of future generations, and how these disruptions are already affecting mental health. At the same time, others felt confident that traditional knowledge could be adapted, and participation in climate action and monitoring were seen as opportunities for empowerment, resilience, and connection to the land, traditional diet, and culture.

Climate change may introduce significant distress by threatening the fabric of Circumpolar communities. Entire coastal communities are displaced by natural disasters (Tran et al., 2021; Wolsko & Marino, 2016), whereas in other regions, individuals are constrained to stay in their communities because of extreme weather or unsafe ice (or ice roads not existing anymore), and thus face exacerbated conflicts in their overcrowded households (Cunsolo Willox et al., 2013a; Harper et al., 2015). The global literature shows that, in the aftermath of natural disasters, family and community relationships can be eroded by stress and division, but not always (Bonanno et al., 2010). The case of the Vietnam NMR illustrates different ways in which community bonds can be mobilized by environmental hazards. This region is strongly affected by climate change, with pervasive consequences on the livelihoods and wellbeing of local ethnic minorities whose subsistence relies on local natural resources (Son & Kingsbury, 2020). Social networks are substantially mobilized to help farming households adapt to unsuccessful harvests related to climate change; this is possible because not all households are affected equally by the environmental changes, and thus those with more capital are in a position to help their neighbors (Son & Kingsbury, 2020). But when all households are simultaneously devastated by floods, there is a decrease in mutual help within communities of NMR, with households individually fencing for themselves. Hence, community resilience is susceptible to environmental disruptions, but when not exceeded, it may buffer the effects of climate change on individual wellbeing.

Across many Circumpolar Peoples, we found reports of fears, worries, sadness, and other distress reactions related directly or indirectly to climate change. Similar reactions have been reported in other parts of the globe, in Indigenous and non-Indigenous Peoples (Cunsolo & Ellis, 2018; Gibson et al., 2019; Johnson et al., 2021; Middleton et al., 2020a, 2020b). Somatic idioms of distress related to climate change have also been reported. For example, in Tuvalu, a Pacific Island nation which is also heavily affected by climate change, somatic experiences such as racing heart, shallow breathing, headache, and muscle tightness were noted alongside emotional and cognitive reactions (Gibson et al., 2019).

Relevant for mental health, we identified determinants of adaptation to climate change. For example, higher income was found to facilitate adaptation (Bunce et al., 2016; Sakakibara, 2010), notably through access to technologies (Brubaker et al., 2011; Ostapchuk et al., 2012). Accordingly, low socioeconomic status has often been associated in the global literature with greater psychological vulnerability to natural disasters (Bonanno et al., 2010; Goldmann & Galea, 2014). As such, socioeconomic inequalities and access to technology may be important targets for enhancing adaptation to climate change and protecting the wellbeing of communities and individuals. Other variables such as sex and age appeared to differentiate the psychological impacts of climate change. Due to the importance of hunting for their identities, young men's sense of self-worth and purpose was particularly affected by climate change (Cunsolo et al., 2020). While older adults have generally been identified as more resilient to natural disasters in the global literature (Bonanno et al., 2010), we found that Elders in the Circumpolar North were significantly preoccupied by the potential impacts of climate change on the identities and livelihoods of their peoples and future generations (Cunsolo et al., 2020; Panikkar & Lemmond, 2020; Petrasek MacDonald et al., 2013). Generational attitudes to climate change have also been reported elsewhere. For example, in the Tuvalu Pacific Island nation, many Elders have expressed a firm intention to remain on their traditional lands, even at the cost of enduring natural hazards and risking their lives, but also a fear for future generations (Gibson et al., 2019). Other subpopulation voices, notably of sexual minorities, were not present in our results.

### Limitations and research gaps

In the 26 articles reviewed, Inuit communities, in particular in Nunatsiavut, predominated, hence not fully representing the vastness and heterogeneity of the Circumpolar North. We could not find research on mental health and climate change in many regions of the Circumpolar North: Inuvialuit Settlement Region (Canada), Norway, Finland, and Greenland. Only one study in Sápmi and one study in Russia were found. Many studies were concentrated in the same communities or regions, particularly in Nunatsiavut which was the focus of 12 articles; although we had limited data to appreciate the extent of overlap between study samples, this imbalance limits the generalizability of our findings. We constrained the current review to peer-reviewed publications in English or French, but grey literature and articles in other languages could hold important data. Only some studies involved distinct groups (e.g., youth, Elders, women). The impacts of climate change on youth, who will have to cope with ongoing climate change, may be particularly critical for further studies considering the importance of land-based skills and livelihoods to their wellbeing and identities, and for cultural continuity in communities. Overall, the lack of representation of distinct groups and communities in the Circumpolar North limits the generalizability of our findings.

More broadly, we found that important determinants of Northern mental health were only beginning to be examined in the climate change literature. As this review has shown, climate change is broadly understood to impact resource availability and predictability, impacting the conditions of individual and collective mental health. Less well developed in the literature are the secondary impacts of environmental changes on social wellbeing, sharing, and feelings of family togetherness fostered by food sharing, preparation, and consumption. Indeed, Inuit in Nunavik report that country foods eaten in the company of others are more physically and socially satisfying than food eaten alone (Fletcher, 2017). These discourses point to how changes in the environment may pose additional, indirect challenges to the wellbeing and ontological security of Circumpolar Peoples.

In Nunavik, food insecurity is a public health priority, with recognized effects on health and child development (Niclasen et al., 2013; Pirkle et al., 2014; Seligman et al., 2009; Thomas et al., 2019). As such, we believe future research should investigate more closely how climate change specifically affects the access to, availability of, preferences for, and quality of country foods, and how these impacts can mediate effects on mental health. Another aspect that warrants consideration is that climate change reduces permafrost coverage (AMAP, 2017; Bell & Brown, 2018; IPCC, 2013, 2018), which is essential for built infrastructures. A case study in Kugluktuk, Nunavut, illustrated how the melting of permafrost is already impacting infrastructures (Prno et al., 2011), and similar issues have been documented in Salluit, Nunavik (Barnard et al., 2015). Their fragilization could worsen pre-existing overcrowding issues, along with their consequences on the mental health of affected families.

In parallel to understanding the consequences of climate change, there is interest in examining the role of community initiatives in promoting adaptation to climate change and mental health. In Australia, land-based programs that reinforce relationships between community members as well as their connection to the natural environment have been put in place with the hope of fostering resilience (Berry et al., 2010). In line with this, there are many examples of Indigenous political, social, and cultural revival, decolonization, and sustainable self-determination efforts across Canada (Grey & Patel, 2015; Kepkiewicz & Dale, 2019). These often involve revitalization of Indigenous foods and ecological knowledge systems (Coté, 2016). However, the possible benefits of adaptation strategies and resilience programs on alleviating mental health impacts of climate changes are not well documented. Considering that anxiety and stress can arise from a disconnection between awareness of climate change and feeling prepared to respond to it (MacKay et al., 2020), the development and dissemination of culturally meaningful adaptation efforts may appreciably participate in fostering community wellbeing.

One area not covered in the current review is the mental health impacts of climate change in individuals leaving their communities and migrating south. Although we touched on the impact of displacement in the aftermath of disasters, there are individuals and families who intentionally leave their traditional homelands to move to larger cities. For example, there is significant Saami migration related to the impacts of climate change on land-based livelihoods (Kelman & Næss, 2019). For these individuals, environmental migration may significantly affect their mental health through upheaval of their cultural identity and social connectedness. This mental health concern was notably reported in the Tuvalu Pacific Island nation (Gibson et al., 2019). Research is needed to examine these effects in Indigenous individuals of the Circumpolar North who migrate to settler cities of the South.

### Implications for clinicians

Because climate change disproportionately impacts the Arctic, and most clinicians are from southern regions where the phenomenon remains mostly marginal, the current findings are significant to better understand the complex multidimensional impacts of climate change on the mental health of Circumpolar Peoples. Health care often seeks transformation through changes in individual behaviors, rather than by addressing broader systemic inequities. Understanding the structural constraints and determinants of inequalities that lead to mental health issues, by broadening the unit of analysis and intervention from individuals to the larger environmental/system-level factors, is a necessary paradigm shift to more fully account for the processes that engender disparities, and for developing preventive policies in response thereto (Gordon & Hunt, 2019; Masuda et al., 2008; Paquin et al., 2020).

Around the Circumpolar North, Indigenous Peoples experience significant environmental and concomitant sociopolitical injustices related to barriers to and disruptions of their traditions, land access, and traditional food systems. These include displacement and dispossession from harvesting grounds and inhabited territories (Colchester, 2004), environmental contamination and degradation associated with industrial development, and deforestation (Finer et al., 2008; Herrmann et al., 2014; Hoover, 2013). Overall, these disruptions add to the broad consequences of climate change (Satterfield et al., 2017; Turner & Clifton, 2009), worsening health inequalities, and the other enduring sociocultural, economic, and political consequences of colonization and globalization (Anaya & Williams, 2001; Colchester, 2004; Hanna & Vanclay, 2013).

Clinicians must be sensitive to health inequities stemming from climate change, which are closely related to colonialism and historical trauma on Indigenous Peoples and cultures. In this context, it is particularly important to recognize that clinicians are often from majority groups, creating an asymmetry within the clinic. Asymmetries in power have significant bearings on Indigenous livelihoods, rights, self-determination, and autonomy, including but not limited to human-driven environmental upheavals. Our awareness of these asymmetries is necessary, both from a perspective of reconciliation and for the clinical understanding of invisible but far-reaching determinants of mental health.

Thus, clinicians must be aware of, and inquire about, the potential effects of environmental changes on patients, their families, and their communities in the Circumpolar North. Naming the contribution of these changes on individual distress can help bring a meaning to it, which can be therapeutic in itself. Clinicians can also provide a space for individuals and families to reflect and comment on the impacts of climate change on their individual and collective lives.

Factors that influence and determine, to various degrees, the impacts of climate change on individuals’ wellbeing were described in this review. Recognizing these factors can help identify at-risk individuals, and they can be targeted by interventions aimed at fostering resilience. By exploring climate change-related experiences with our patients, such as food insecurity, boredom, a disrupted sense of purpose, housing problems, social tensions, or concerns for the future, we may be better positioned to assist efforts in adapting to climate change. Cunsolo et al. (2020) highlighted that losses and worries related to climate change in the Circumpolar North can be experienced as a form of grief. Overall, many of the mental health consequences and pathways identified in this review fit with the notion of ecological grief. This form of grief is felt in response to “experienced or anticipated ecological losses, including the loss of species, ecosystems and meaningful landscapes” (Cunsolo & Ellis, 2018). It can emerge from physical ecological losses, such as the inability to travel on the ice or to sustain reindeer herding, but also from disruptions of environmental knowledge systems and senses of identity, as well as anticipated future losses. The mental health impacts of climate change in the Circumpolar North are also consistent with the concept of eco-anxiety, defined as worries and fears related to climate change, including about one own's wellbeing, but also that of animals, nature, and future generations (Clayton et al., 2017; Ojala et al., 2021).

The emotional reactions, appraisals of, and responses to climate change highlighted in the current review, as well as in the Tuvalu nation (Gibson et al., 2019) for example, and among Australian farmers (Cunsolo & Ellis, 2018), can be conceptualized as expressions of ecological grief and anxiety. But in contrast to the loss of a loved person, ecological grief and worry is generally unacknowledged and unrecognized, and it involves an “ambiguous loss,” in the sense that it stems from dynamic losses that may never end nor lead to a sense of closure (Cunsolo & Ellis, 2018). Consequently, we believe that, as researchers and clinicians, we have a responsibility to engage with communities, environmental stakeholders, lodging, and other local organizations and governments to recognize these losses, and to innovate and improve our approaches to the associated needs.

## Conclusion

The impacts of climate change on mental health are felt across the Circumpolar North. For Circumpolar Peoples whose cultures, sense of self, and ways of living are closely connected to the land, environmental upheavals related to climate change threaten cultural, social, and physical determinants of health and wellbeing. As a result, anxious and depressive reactions, anger, and powerlessness are reported across Northern communities, but also optimism, resilience, and innovative adaptation strategies. Conceptions of wellbeing and mental health differ among peoples and generations, along with the determinants of wellbeing and the environmental changes that influence them. More research is needed to better understand how communities and regions are distinctly affected by climate change (e.g., in Nunavik), with the aim of co-developing locally adapted strategies for adaptation and resilience. Gaps in scientific literature must be addressed through ongoing co-creation of knowledge with communities from all Circumpolar regions. Understanding vulnerability and resilience factors is crucial for mental health clinicians hoping to meet emerging needs under a changing climate. Clinicians and researchers can also play a role in advocating for policies sensitive to the climate–mental health relationship in the Arctic and elsewhere.

## Supplementary Material

Supplementary material
