# Supplementary material for: Climate change and Indigenous mental health in the Circumpolar North: A systematic review to inform clinical practice
Source: Transcult Psychiatry. 2022 Jan 6;59(3):312–36. doi: 10.1177/13634615211066698 (PMC9160950; doi:10.1177/13634615211066698)
Supplement: Supplementary material [file sj-docx-1-tps-10.1177_13634615211066698.docx]

Climate change and mental health in the circumpolar North: a systematic review to inform clinical practice

SUPPLEMENTARY FILE

CONTENT

Table S1 – PRISMA checklist

Table S2 – Search vocabulary

Table S3 - Reported emotional reactions to climate change among Circumpolar Peoples

| Table S1. PRISMA checklist | | | |
| --- | --- | --- | --- |
| **Section/topic** | **#** | **Checklist item** | **Reported on page #** |
| **TITLE** | | |  |
| Title | 1 | Identify the report as a systematic review, meta-analysis, or both. | 1 |
| **ABSTRACT** | | |  |
| Structured summary | 2 | Provide a structured summary including, as applicable: background; objectives; data sources; study eligibility criteria, participants, and interventions; study appraisal and synthesis methods; results; limitations; conclusions and implications of key findings; systematic review registration number. | 1 |
| **INTRODUCTION** | | |  |
| Rationale | 3 | Describe the rationale for the review in the context of what is already known. | 2,3,4 |
| Objectives | 4 | Provide an explicit statement of questions being addressed with reference to participants, interventions, comparisons, outcomes, and study design (PICOS). | 4 |
| **METHODS** | | |  |
| Protocol and registration | 5 | Indicate if a review protocol exists, if and where it can be accessed (e.g., Web address), and, if available, provide registration information including registration number. | N/A |
| Eligibility criteria | 6 | Specify study characteristics (e.g., PICOS, length of follow-up) and report characteristics (e.g., years considered, language, publication status) used as criteria for eligibility, giving rationale. | 4,5 |
| Information sources | 7 | Describe all information sources (e.g., databases with dates of coverage, contact with study authors to identify additional studies) in the search and date last searched. | 4 |
| Search | 8 | Present full electronic search strategy for at least one database, including any limits used, such that it could be repeated. | Supplementary file |
| Study selection | 9 | State the process for selecting studies (i.e., screening, eligibility, included in systematic review, and, if applicable, included in the meta-analysis). | 5 |
| Data collection process | 10 | Describe method of data extraction from reports (e.g., piloted forms, independently, in duplicate) and any processes for obtaining and confirming data from investigators. | 5,6 |
| Data items | 11 | List and define all variables for which data were sought (e.g., PICOS, funding sources) and any assumptions and simplifications made. | 5,6 |
| Risk of bias in individual studies | 12 | Describe methods used for assessing risk of bias of individual studies (including specification of whether this was done at the study or outcome level), and how this information is to be used in any data synthesis. | N/A |
| Summary measures | 13 | State the principal summary measures (e.g., risk ratio, difference in means). | N/A |
| Synthesis of results | 14 | Describe the methods of handling data and combining results of studies, if done, including measures of consistency (e.g., I^2^) for each meta-analysis. | 6 |
| Risk of bias across studies | 15 | Specify any assessment of risk of bias that may affect the cumulative evidence (e.g., publication bias, selective reporting within studies). | N/A |
| Additional analyses | 16 | Describe methods of additional analyses (e.g., sensitivity or subgroup analyses, meta-regression), if done, indicating which were pre-specified. | N/A |
| **RESULTS** |  |  |  |
| Study selection | 17 | Give numbers of studies screened, assessed for eligibility, and included in the review, with reasons for exclusions at each stage, ideally with a flow diagram. | Figure 1 |
| Study characteristics | 18 | For each study, present characteristics for which data were extracted (e.g., study size, PICOS, follow-up period) and provide the citations. | Table 1 |
| Risk of bias within studies | 19 | Present data on risk of bias of each study and, if available, any outcome level assessment (see item 12). | N/A |
| Results of individual studies | 20 | For all outcomes considered (benefits or harms), present, for each study: (a) simple summary data for each intervention group (b) effect estimates and confidence intervals, ideally with a forest plot. | N/A |
| Synthesis of results | 21 | Present results of each meta-analysis done, including confidence intervals and measures of consistency. | 6–17 |
| Risk of bias across studies | 22 | Present results of any assessment of risk of bias across studies (see Item 15). | N/A |
| Additional analysis | 23 | Give results of additional analyses, if done (e.g., sensitivity or subgroup analyses, meta-regression [see Item 16]). | N/A |
| **DISCUSSION** |  |  |  |
| Summary of evidence | 24 | Summarize the main findings including the strength of evidence for each main outcome; consider their relevance to key groups (e.g., healthcare providers, users, and policy makers). | 17–22 |
| Limitations | 25 | Discuss limitations at study and outcome level (e.g., risk of bias), and at review-level (e.g., incomplete retrieval of identified research, reporting bias). | 19 |
| Conclusions | 26 | Provide a general interpretation of the results in the context of other evidence, and implications for future research. | 23 |
| **FUNDING** |  |  |  |
| Funding | 27 | Describe sources of funding for the systematic review and other support (e.g., supply of data); role of funders for the systematic review. | 24 |

| Table S2. Controlled vocabulary and free queries used in each database | |
| --- | --- |
| **Free queries** | |
| Single term queries : Inuit OR Inuk OR Inuq OR Eskimo* OR Kalaallit* OR Inupia* OR Aleut* OR Nunangat OR Nunavik OR Nunavut OR Nunatsiavut OR Inuvialuit OR Sami OR Saami OR Yup’ik* OR Yupik* OR Yakut* OR Sakha OR Aleut* OR Unanga* OR Netsilik OR Alutiiq OR Sugpiaq* OR Chugach | |
| **OR** | |
| Double term queries (Location and Population) : Location: North* OR Canada OR Arctic OR Alaska OR Circumpolar OR Greenland OR Denmark OR Northwest Territories OR Norway OR Russia OR Sweden OR Finland OR Iceland OR polar OR Yukon OR Kodiak OR Chukotka OR Siberia  AND  Population: Indigenous OR Aboriginal OR “Native Nation” OR “Native Nations* | |
| **AND** | |
| “Climate change” OR “global warming” OR heating OR “greenhouse effect” OR “temperature change” OR “water resources” OR flood* OR “extreme weather” OR “sea levels” OR “sea level” | |
| **AND** | |
| Mental OR well-being OR wellness OR resilien* OR grief OR Cultur* OR boredom OR self-esteem OR distress OR stress OR suicide* OR depressi* OR happiness OR emotion* OR protective factor* OR “adaptive capacity” OR “adaptive capacities” OR “adaptive ability” OR “adaptive abilities” OR psycholog* OR psychiatr* OR violence OR “substance abuse” OR addictions | |
| **Controlled vocabulary (Thesaurus)** | |
| **Database** | **Thesaurus** |
| PubMed and Cochrane | (”Inuits” OR “Alaska natives” OR "Arctic Regions/epidemiology") AND "Psychiatry and Psychology Category" AND (“Climate change” OR “greenhouse effect”) |
| PsychInfo (PsyNet) | Climate change AND (Inuit OR Alaska Natives) |
| Web of Science | No thesaurus |
| Embase | ('Eskimo-Aleut people'/exp OR 'Alaska Native'/exp OR 'Canadian Aboriginal'/exp OR 'First Nation'/exp) AND ( 'greenhouse effect'/exp OR 'climate change'/exp) AND ('mental health'/exp OR 'behavior'/exp OR 'resilience'/exp OR 'mental function'/exp OR 'cultural anthropology'/exp OR 'mental disease'/exp OR 'psychology'/exp OR 'psychiatry'/exp OR 'social problem'/exp) |
| GeoBase | indigenous population AND (climate change OR environmental change OR greenhouse effect) AND mental health |
| CINAHL | indigenous population AND (climate change OR environmental change OR greenhouse effect) AND mental health |

| Table S3. Reported emotional reactions to climate change among Circumpolar Peoples | |
| --- | --- |
| Depressive reactions | Boredom, suicide ideation, depression, helplessness, sadness, grief (people and environment), distress, loneliness, isolation, restlessness, disappointment |
| Loss | Loss of identity, purpose, feeling of having a handicap, feeling of loss about the environment. |
| Anxious reactions | Stress (about changes, feeling stuck, predators), fear and worry (about safety, being relocated, decline of animal population because of climate change), anxiety (because of vulnerability, unpredictability of hunts) |
| Other reactions | Anger, frustration, helplessness, upset |
